# CCN1-induced senescence and dysfunction of umbilical cord blood endothelial colony-forming cells in the pathogenesis of preeclampsia

**DOI:** 10.3389/fendo.2026.1930702

**Published:** 2026-08-28

**Authors:** Yuyang Ma, Fenglian Yang, Yu Wang, Yingya Li, Yanping Jia, Jie Wang, Kunming Li, Liyan Duan

**Affiliations:** 1 Obstetric, Gynecology and Reproductive Medicine Center, Shanghai Tenth People’s Hospital, Tongji University School of Medicine, Shanghai, China; 2 Center for Reproductive Medicine and Obstetric and Gynecology, Nanjing Drum Tower Hospital, Affiliated Hospital of Medical School, Nanjing University, Nanjing, China; 3 Department of Obstetrics and Gynecology, Shanghai First Maternity and Infant Hospital, Tongji University School of Medicine, Shanghai, China

**Keywords:** CB-ECFCs, CCN1, cellular senescence, notch pathway, preeclampsia

## Abstract

**Introduction:**

Maternal vascular dysfunction and impaired placental angiogenesis are key features of preeclampsia (PE). Umbilical cord blood-derived endothelial colony-forming cells (CB-ECFCs) contribute to fetal endothelial repair and vascular network formation, but their phenotype in PE remains incompletely understood.

**Methods:**

We investigated whether the matricellular protein CCN1 is associated with CB-ECFC senescence and dysfunction. CB-ECFCs were isolated from pregnancies complicated by PE (n = 8) and healthy pregnancies (n = 9). Cellular senescence was assessed by SA-β-galactosidase staining, qPCR, and Western blotting of senescence-associated markers and senescence-associated secretory phenotype factors. Control-derived CB-ECFCs were exposed to recombinant CCN1, with or without the gamma-secretase inhibitor DAPT, and senescence-associated proteins, cleaved Notch1, the redox-responsive protein HO-1, the inflammatory factor TNF-α, and the DNA damage-response marker gamma-H2AX were examined.

**Results:**

PE-derived CB-ECFCs showed reduced colony yield, increased senescence-associated markers, and higher secreted and cell-associated CCN1 expression. Recombinant CCN1 increased senescence-associated markers, cleaved Notch1, HO-1, TNF-α, and γ-H2AX and impaired CB-ECFC metabolic activity, migration, and tube formation. DAPT attenuated selected molecular changes and partially restored tube formation but not wound closure.

**Discussion:**

These findings identify increased CCN1 as a feature of PE-derived CB-ECFCs and support an association between CCN1 exposure, Notch1 signaling, oxidative stress, and CB-ECFC senescence and dysfunction. The CCN1/Notch1 axis may represent a potential mechanism contributing to fetoplacental endothelial dysfunction in PE.

## 1 Introduction

Preeclampsia (PE) is a multifactorial pregnancy-specific hypertensive disorder characterized by the onset of hypertension and proteinuria after 20 weeks of gestation (1). It affects 2%–8% of pregnancies worldwide (2). Although its etiology remains incompletely unclear, maternal endothelial dysfunction and abnormal placental vascular remodeling may play a role in the pathogenesis of PE and may persist postpartum (3, 4). Defective placentation may lead to placental ischemia and an imbalance between pro-angiogenic and anti-angiogenic factors, thereby promoting endothelial injury (5). Excessive release of anti-angiogenic factors into the maternal circulation can cause systemic endothelial damage. Maintaining endothelial cell function is crucial for preventing pan-vascular disease progression and addressing vascular dysfunction caused by diverse etiologies and physicochemical factors, as endothelial homeostasis imbalance drives these pathological processes (6). Clarifying the cellular mechanisms underlying endothelial dysregulation in PE may therefore help identify new therapeutic targets.

Endothelial colony-forming cells (ECFCs), a highly proliferative subtype of endothelial progenitor cells (EPCs), possess the capacity to differentiate into endothelial-phenotype cells and form functional vascular networks *in vivo* (7). Functionally competent umbilical cord blood endothelial colony-forming cells (CB-ECFCs) play a pivotal role in placental vascular development and endothelial repair. Previous studies have demonstrated that PE is associated with both quantitative reductions in ECFCs and qualitative impairments of their migratory and angiogenic capacities (8–11). These pathological alterations may contribute to aberrant placental angiogenesis, endothelial dysfunction, and subsequent impairment of fetoplacental oxygen and nutrient exchange (12). However, the molecular mechanisms underlying ECFC dysfunction in PE remain poorly understood.

Cellular senescence is an important driver of endothelial dysfunction. Senescent EPCs show reduced proliferative and reparative capacity, thereby limiting their contribution to endothelial regeneration and vascular repair (13). According to Simoncini et al. (2022), senescence-associated secretory phenotype(SASP) expression increased in patients with severe atherosclerotic cardiovascular disease (ASCVD) due to increased ECFC senescence, which impacted the capacity of ECFC to repair vascular endothelial cells (14), and senescence-associated EPC dysfunction may contribute to vascular susceptibility during aging (15). This phenomenon is further illustrated by mycophenolic acid-induced cellular senescence in CB-ECFCs, which leads to marked reductions in both proliferative capacity and angiogenic potential (16). Importantly, developmental abnormalities in CB-ECFCs have been mechanistically linked to vascular pathogenesis. Clinical observations have demonstrated that the diminished CB-ECFC frequency in extremely preterm neonates correlates with impaired pulmonary vascular maturation, suggesting a critical role in developmental angiogenesis (17). These findings suggest that accelerated CB-ECFC senescence in PE may reduce ECFC number and function, ultimately compromising placental vascular network formation and endothelial repair.

CCN1, also known as CYR61, is a member of the CCN family of extracellular matrix-associated matricellular proteins.CCN1 interacts with multiple signaling molecules and receptors, including integrins, bone morphogenetic proteins, vascular endothelial growth factors (VEGF), Wnt, and Notch (18). A large number of studies have demonstrated that CCN1 can promote cellular senescence through multiple mechanisms (18, 19), and increased CCN1 expression in the placenta can lead to the onset of PE (20). Previous studies have demonstrated that CCN1 and CCN3 induce trophoblast cells senescence by activating the Notch pathway (21). Recent studies show that ROS-associated oxidative stress damages endothelial cell survival and vascular structures, impairs endothelial progenitor cell angiogenesis and vascular organoid development, and thereby suggests a broader role for oxidative stress in cellular senescence (22, 23) Notably, Notch signaling is dynamically active in both maternal and fetal endothelial cells, as well as trophoblast populations, throughout placental development—from early implantation to term pregnancy (24). This pathway serves as a critical regulator of angiogenesis and vascular remodeling (25), further underscoring its multifaceted role in placental biology. Moreover, studies have shown that Notch1 expression is significantly increased in EPCs of patients with PE compared to healthy controls (26). Based on this, we hypothesized that CCN1 expression may be linked to CB-ECFC senescence and dysfunction through Notch1-associated oxidative stress.

The functional alterations of CB-ECFCs, the expression pattern and role of CCN1 in PE remain incompletely understood. Therefore, this study investigated CCN1 expression and cellular senescence in CB-ECFCs from PE pregnancies and examined the effects of exogenous CCN1 on CB-ECFC function. We further explored whether Notch1 signaling, oxidative stress, and DNA damage mediated CCN1-induced CB-ECFC senescence and dysfunction.

## 2 Materials and methods

### 2.1 Cell culture and reagents

#### 2.1.1 Isolation, culture, and identification of CB-ECFCs from PE and normal pregnancies

The study was approved by the Ethics Committee of Shanghai Tenth People’s Hospital affiliated with Tongji University (SHSY-IEC-6.0/25KY9/P01). Umbilical cord blood-derived endothelial colony-forming cells (CB-ECFCs) were isolated from patients with PE (n=8) and healthy controls (n=9) using established protocols (27, 28). Briefly, cord venous blood was collected post-delivery, and mononuclear cells were isolated via density gradient centrifugation (Ficoll Plus, GE Healthcare #17-1440-3; 400 ×g, 40 min). CB-ECFCs were maintained in endothelial cell growth medium (EGM)-2medium (Lonza, basal, CC-3156) supplemented with 10% fetal bovine serum (FBS)(Gibco, A5669701), 1% penicillin/streptomycin (P/S) (Thermo Fisher,15140122), and endothelial growth factors (EGM^®^-2 SingleQuots^®^ Supplements, Lonza, CC-4176) at 37°C with 5% CO_2_. Only early-passage(passages 3-7) CB-ECFCs were used in this study. Within each independent experiment, the treatment groups and their corresponding controls were derived from the same isolation batch and examined at the same or closely matched passage stage to minimize potential confounding by replicative senescence.

#### 2.1.2 Intervention studies

CB-ECFCs from controls were stimulated with recombinant human CCN1 protein (Novoprotein, #CB98; 0–500 ng/mL) or the γ-secretase inhibitor DAPT (N-[N-(3,5-difluorophenacetyl-L-alanyl)]-S-phenylglycine t-butyl ester; Sigma, D5942). Unless otherwise specified, the duration of all stimulation treatments was set to 24 h. To minimize serum-induced variability, treatment experiments utilized EMG-2 with 2% FBS, 1% P/S, and growth factors under standard culture conditions (37°C, 5% CO_2_).

Given the limited availability of CB-ECFCs collected from patients and the fact that cellular characteristics may alter with increasing passage numbers, the 9 samples from the healthy control group were randomly divided into two groups: one for CCN1 concentration-gradient stimulation experiments (n = 5) and the other for DAPT rescue experiments (n = 4).

### 2.2 Senescence-associated β-galactosidase staining

Cellular senescence was assessed using the Senescence β-Galactosidase Staining Kit (Beyotime Institute of Biotechnology, Cat# C0602). This assay was used to evaluate senescence in CB-ECFCs from PE and healthy control pregnancies and to examine changes in senescence after exogenous CCN1 stimulation with or without DAPT treatment. CB-ECFCs at passage 5–6 were washed twice with PBS and subsequently fixed using the supplied fixation agent for 15 minutes at room temperature. After removing the fixative and washing with PBS, CB-ECFCs were incubated in a staining solution containing X-gal in a pH 6.0 buffer for 20 hours at 37°C in a non-CO2 incubator. Bright-field microscopy images were acquired from three randomly selected fields per experimental group, and quantitative values were averaged across these fields. Senescence quantification was performed using Image-Pro Plus software (v6.0).

### 2.3 Quantitative real-time polymerase chain reaction

Total RNA was extracted using TRIzol reagent (Ambion,15596-026) following themanufacturer’s protocol. cDNA was synthesized from 2 μg of total RNA in a 20-μl reaction volume using 5× All-In-One RT MasterMix (abm, G492). Quantitative real-time PCR was performed with 2× SYBR Green Master Mix (Vazyme) on an ABI Prism 7300 sequence detection system (Applied Biosystems, Foster City, USA). The constitutively expressed Actin gene was used as an endogenous control for normalization. Relative mRNA expression levels were calculated using the comparative Ct (2−ΔΔCt) method and outliers were detected and excluded using the ROUT method (Q = 1%) in GraphPad Prism. Primer sequences are provided in **Supplementary Table 1**.

### 2.4 Protein extraction

#### 2.4.1 Secretory protein extraction

Cells were cultured in complete medium supplemented with serum until reaching 70% confluence. The serum-containing medium was then aspirated and replaced with serum-free medium. After 48 hours of incubation (at 85–90% confluence), the serum-free medium was collected and subjected to sequential centrifugation at 800 × g for 5 minutes at 4°C to remove cellular debris. This centrifugation step was performed twice to get secretory protein. The secretory protein was concentrated using 6 mL centrifugal filter units with a 3 kDa molecular weight cutoff membrane (Thermo Scientific™ Pierce™, Cat# 88515) according to the manufacturer’s instructions. The protein concentration was determined using the BCA Protein Assay kit (Beyotime, Jiangsu, China, #P0011). After that, the concentrated protein samples were aliquoted and stored at –80°C until further analysis.

#### 2.4.2 Cell protein extraction

RIPA buffer containing protease inhibitors (Roche, Branford, CT, USA, #11697498001) and a phosphatase inhibitor (Sigma-Aldrich, #P5726) was used to extract the protein from the cells (passage 4-5). High-speed centrifugation was performed at 4°C to extract the supernatant. The BCA Protein Assay kit (Beyotime, Jiangsu, China, #P0011) was used to measure the protein concentration. Protein samples were aliquoted and stored at -80°C until further use.

### 2.5 Western blot

20 μg protein was separated by SDS–PAGE and transferred to PVDF membranes(Merck Millipore, Darmstadt, Germany, #03010040001). The membranes were blocked with 5% skimmed milkfor 1 hour at room temperature and then incubated overnight at 4°C with primary antibody. The secondary antibody was incubated for one hour at room temperature (for antibody information, see **Supplementary Table 2**). The ECL chemiluminescence kit (Beyotime Biotechnology) was used to detect protein signals according to the manufacturer’s instructions. Images were acquired using the Chemidoc XRS+ imaging system (BioRad, Feldkirchen, Germany). Densitometric analysis was performed with ImageJ 2x (Rawak Software Inc., Germany). For target proteins and Actin, the signal intensity of individual discrete bands was quantified. For Ponceau S staining, the total integrated density of the entire lane was measured to represent total protein loading. Protein expression levels were subsequently normalized to either Actin or total protein (Ponceau S) as indicated in the figure legends.

### 2.6 Immunofluorescence staining

After fixing with 4% paraformaldehyde for 15 minutes, CB-ECFCs at passage 4–5 werepermeabilized with 0.1% Triton X-100 in PBS for 5 minutes and blocked with 5% BSA in PBS for 1 hourat room temperature. The cells were incubated with primary antibodies for 1 hour at room temperature, followed by fluorescence-conjugated secondary antibodies to detect primary antibody binding (**Supplementary Table 3**). DAPI (DAPI, 1µg/ml, Sigma Aldrich, St. Louis, USA) was used for 10 minutes at room temperature for nuclear staining. A confocal fluorescence microscope (Nikon eclipse Ts2R) was used to view the slides after they had been covered with Mowiol (Sigma-Aldrich, China).

### 2.7 3-(4,5-dimethylthiazol-2-yl)-2,5-diphenyltetrazoliumbromide (MTT) assay

Cell proliferation was assessed using the MTT assay. Briefly, cells at passage 3-4 (3.5×10^3^/100 μL) were plated on flat-bottom 96-well plates and allowed to adhere overnight. The cells were then stimulated with different concentrations (0, 10 ng/mL, 50 ng/mL, 100 ng/mL, 500 ng/mL) of CCN1 for 24 and 48 hours. MTT (final concentration 5 mg/mL) was added to each well after CCN1 stimulation, followed by 4 h of incubation at 37°C in the dark. Each well was treated with 100 μL of isopropanol to dissolve the formazan crystals, after which the absorbance at 570 nm was measured using a microplate reader (Infinite M200 PRO, Tecan, Sweden).

### 2.8 Cell migration

To investigate the effects of CCN1 on CB-ECFC migration, wound healing assays were conducted as follows. CB-ECFCs from healthy controls at passage 3–4 were seeded at 3.5×10^5 per well in 6-well plates and cultured overnight. Subsequently, a sterile pipette tip was used to create a linear wound by scraping the cell monolayer. The wells were then rinsed thrice with PBS to remove the detached cells. Following this, recombinant human CCN1 at a concentration of 100 ng/ml or 500 ng/ml (with or without DAPT) was added to the respective wells. Images of wound closure were captured at 0 h (immediately after wounding) and 8 h post-incubation.

### 2.9 Angiogenesis assay

In each well of the 96-well plate, 50 μl of pre-cooled 10 mg/ml Matrigel-Matrix (Life Sciences, 56255) was pipetted. The plate was incubated at 37°C for 30 min to enable the matrix gel to solidify. Subsequently, 100 μl of CB-ECFC (at passage 3-4) suspension (3×10^4^ cells) was added to each well and incubated for 6 h. After incubation, images of tube morphology were taken by an inverted microscope (Leica). ImageJ2x (Rawak Software Inc., Germany) plug-in “Angiogenesis Analyzer” was used to analyze the results of the tube formation experiment.

### 2.10 Statistical analysis

G Power software was used to determine the sample size for this study.

All statistical analyses were conducted in GraphPad Prism. Definitive outliers were removed via the ROUT test with Q = 1%. The Shapiro–Wilk test was utilized to assess data normality, while the F-test was employed to evaluate variance homogeneity (α = 0.05). For datasets conforming to normal distribution and equal variance, unpaired two-tailed Student’s t-test was used for comparisons between two groups; one-way analysis of variance (ANOVA) coupled with Tukey’s *post-hoc* test was applied for multiple-group comparisons. Non-parametric tests were selected for samples failing normality or homoscedasticity assumptions. All quantitative data are expressed as mean ± standard deviation (mean ± SD). Two-tailed P values were adopted to determine statistical significance, with thresholds defined as *P < 0.05, **P < 0.01, ***P < 0.001, and ****P < 0.0001.

## 3 Results

### 3.1 Clinical characteristics and characterization of ECFCs

**Table 1** shows the clinical characteristics of pregnant patients who provided umbilical cord bloodfor CB-ECFC isolation. A total of 17 pregnant women were included (Control, n=9; PE, n=8). Maternal age, BMI, spontaneous conception rate, cesarean delivery rate and baby gender did not differ significantly between groups (p > 0.05). As expected, systolic and diastolic blood pressure were significantly higher in PE group than in the control group (p < 0.001). The fetal weight in the PE group was lower than that in the control group (p=.0011), possibly because the fetuses in the control group were delivered earlier. The 24-h urinary protein quantitation could not be analyzed, as urine samples of healthy controls were not routinely collected. CB-ECFCs isolated from all study participants were cultured under standardized conditions, and cobblestone-like colonies emerged within 5–20 days (29). Cell identity was confirmed by dual validation using flow cytometry and immunofluorescence staining, revealing a CD31^+^/CD45^-^/CD133^-^ immunophenotype that aligns closely with previously published findings (30, 31) (**Supplementary Figure 1**). Flow cytometry analysis revealed that the vast majority of the isolated cells were CD45-negative. Although a minor fraction (~8%) of CD45-positive residual hematopoietic cells was observed at the early passage, these non-adherent and non-proliferating cells were depleted during subsequent media changes and serial passaging, ensuring a highly pure ECFC population for all downstream functional analyses. Notably, there was a substantial decrease in cord blood ECFC colonies in PE compared to controls (3.0 ± 1.1 (control) vs. 1.5 ± 0.9 (PE)) (p = 0.009), indicating reduced colony-forming capacity in PE-derived CB-ECFCs.

**Table 1:** Clinical and demographic data for patients with preeclampsia and healthy controls who provided samples in this study.

Variable	Healthy control (n=9)	Preeclampsia (n=8)	P value
Maternal age (y)	31.3 ± 3.8	34.0 ± 2.1	0.091
Maternal BMI (kg/m2)	23.1 ± 3.5	25.3 ± 5.6	0.358
Conception, spontaneous-n(%)	1(11.1%)	2(25%)	0.576
Gestational SBP, pre-delivery (mm Hg)	118.7 ± 9.9	145.3 ± 11.3	<0.001*
Gestational DBP, pre-delivery (mm Hg)	70.1 ± 5.8	84.9 ± 5.4	<0.001*
24-Hour Urinary Protein Quantitation(mg)	/	1501.3 ± 368.5	/
ALT	13.8 ± 3.6	11.6 ± 6.7	0.412
AST	20.9 ± 6.1	18.7 ± 8.0	0.536
Platelet Count	215.0 ± 49.0	233.3 ± 72.5	0.548
Gestational age at delivery (wk)	38.6 ± 1.9	37.9 ± 1.1	0.074
Preterm birth-n(%)	1(11.1%)	1(12.5%)	>0.99
Caesarean delivery- n (%)	6(66.7%)	5(62.5%)	>0.99
Baby gender, male- n (%)	6(66.7%)	3(37.5%)	0.347
Birth weight (g)	3343.3 ± 409.1	3085.0 ± 268.0	0.011*
Gestational age-adjusted birth weight Z-score	0.42 ± 0.36	0.11 ± 0.88	0.370
Colony numbers (per 50 million PBMCs)	3.0 ± 1.1	1.5 ± 0.9	0.009*
Days to first appearance of colonies	16.9 ± 1.5	18.0 ± 1.5	0.155

BMI, body mass index; DBP, SBP, diastolic and systolic blood pressure; PBMC: peripheral blood mononuclear cells; ALT, Alanine Aminotransferase; AST, Aspartate Aminotransferase.

Data are mean ± SD or number (percent). *p<0.05 using t tests (nonparametric test) and Chi-square tests.

### 3.2 Cellular senescence in CB-ECFCs of PE and healthy control patients

**Figure 1:**
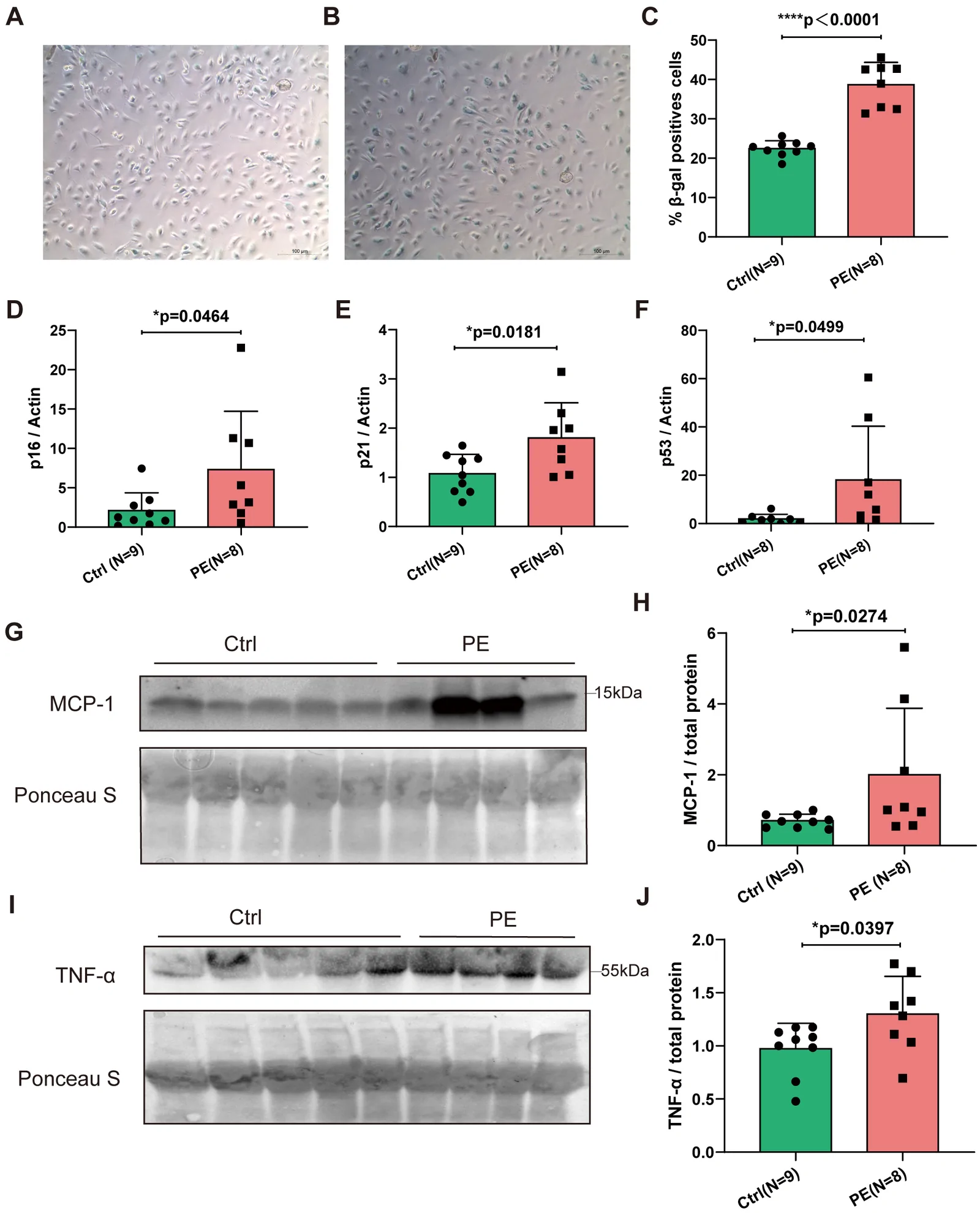
The senescence levels of CB-ECFCs in PE patients and healthy controls. SA-β-Gal staining for CB-ECFCs from patients with PE and healthy controls **(A–C)**. qPCR analysis of mRNA levels of senescence associated factors p16, p21 and p53 **(D–F)**. Western blot analysis of SASP-related factors MCP-1 and TNF-α in CB-ECFCs from the two groups, with a representative region of Ponceau S staining shown as loading control and quantification performed on the entire lane **(G–J)**. Significance for all data was determined by Student’s t-test. Data are presented as mean ± S.D (Note that outliers were excluded in panel F, and the final sample size for the control group was n = 8). *p < 0.05, ***p < 0.0001. Ctrl represents the healthy control group, PE represents the PE patient group.

SA-β-Gal staining results showed that CB-ECFCs from PE patients had significantly increased senescence compared with controls (p<0.001, **Figures 1A–C**). The mRNA expression levels of senescence-associated markers p16, p21, and p53 were also significantly increased (**Figures 1D–F**). Western blotting analysis further showed increased secretion of SASP-related secretory proteins TNF-α and MCP-1 in the culture supernatant (p=0.0274 and p=0.0397, **Figures 1G–J**).

### 3.3 CCN1 expression increased in ECFCs from PE patients

**Figure 2:**
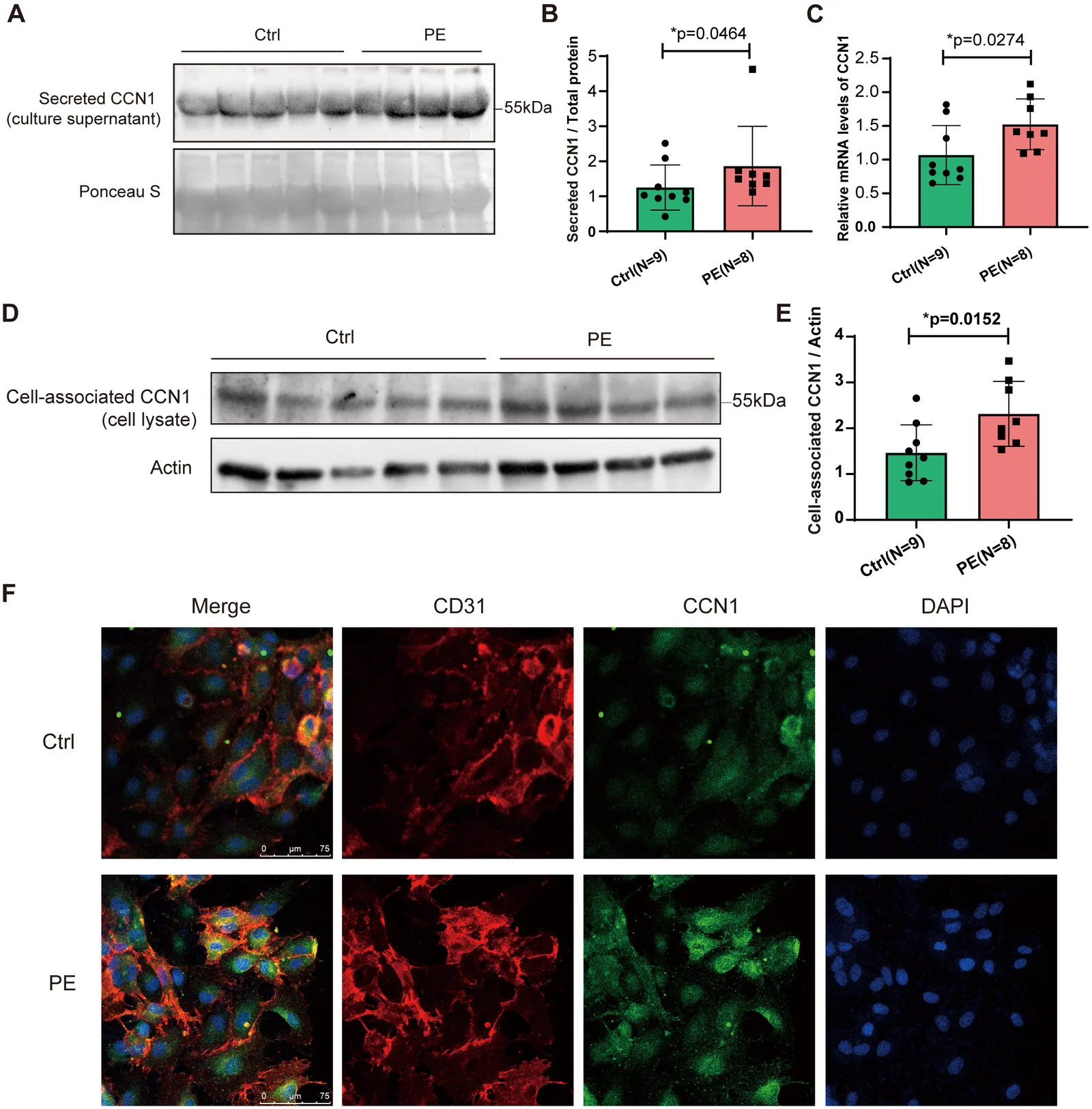
CCN1 expression in CB-ECFCs of PE patients and healthy controls. Western blot analysis of secreted CCN1 in CB-ECFC culture supernatants from the two groups, ponceau S staining was used as the loading control for culture supernatant samples because β-actin is not an appropriate loading control for secreted proteins. **(A, B)**. qPCR **(C)** and western blot analysis **(D, E)** of membrane-bound CCN1 in CB-ECFCs from the two groups. Immunofluorescence staining of DAPI (blue, nuclear counterstain), CD31 (red, CB-ECFC marker) and CCN1 (green) in the two groups; merged images show co-localization signals. Scale bar = 75 μm **(F)**. Significance for all data was determined by Student’s t-test. Data are presented as mean ± S.D. *p < 0.05. Ctrl represents the healthy control group, PE represents the PE patient group.

Compared to the healthy control group, secreted CCN1 was significantly elevated in the culture supernatant of CB-ECFCs from PE patient samples (p=0.0464, **Figures 2A, B**). Both protein and mRNA expression levels of membrane-bound CCN1 were upregulated in CB-ECFCs from PE patients compared to controls (p=0.0274 and p=0.0152, respectively; **Figures 2C–E**). These findings suggest a potential role for CCN1 in mediating CB-ECFC dysfunction under PE conditions. To further characterize CCN1 localization, immunofluorescence staining was performed. The results showed that in both groups, CCN1 expression was consistently localized to the cell membrane of CB-ECFCs, suggesting a potential role for CCN1 in membrane-related biological processes of CB-ECFCs (**Figure 2F**).

### 3.4 CCN1 inhibits proliferation, angiogenesis, and migration of CB-ECFCs

**Figure 3:**
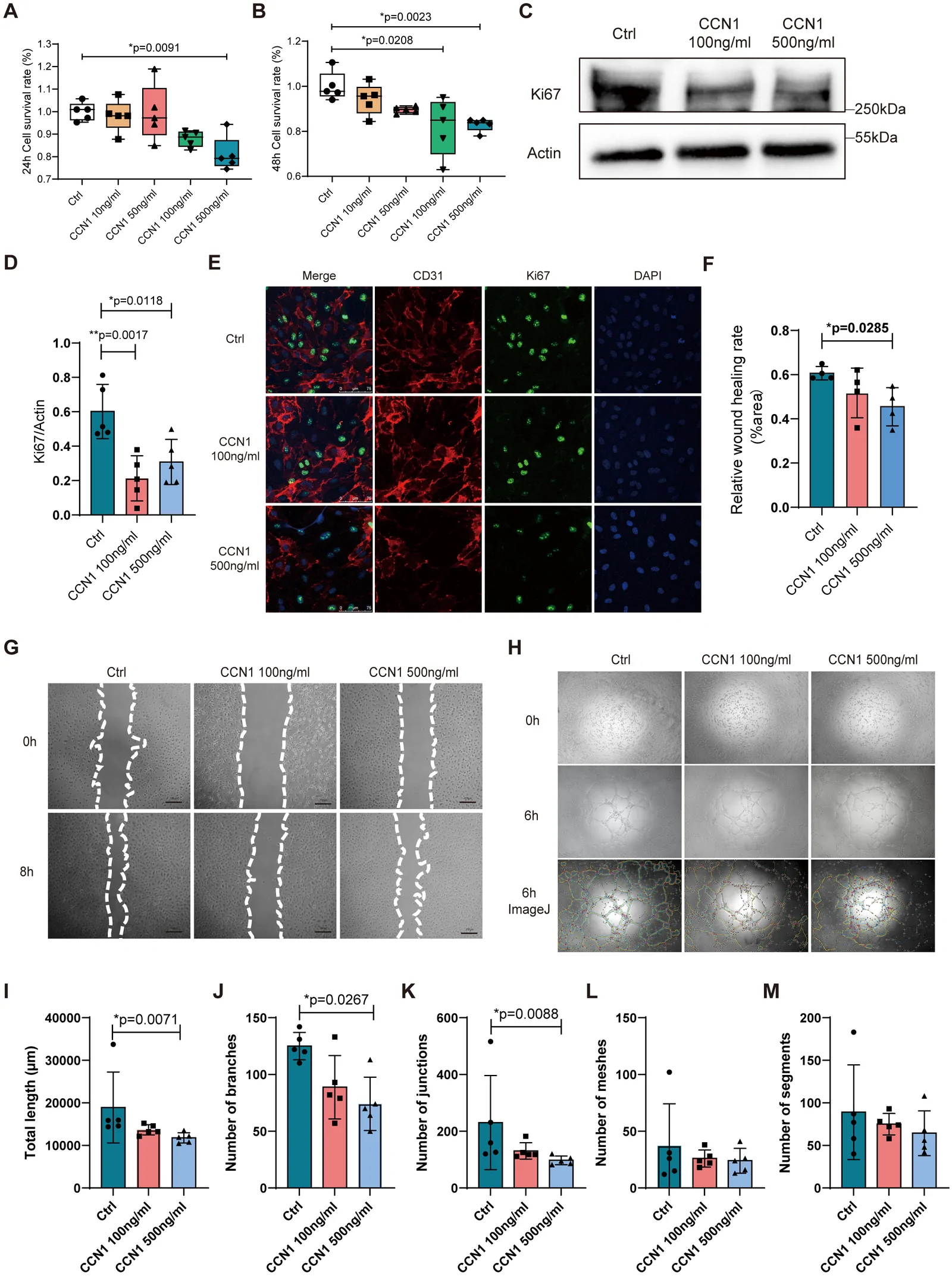
The impact of exogenous CCN1 at different concentrations on the function of CB-ECFCs isolated from normal pregnancies (N = 5). MTT assay for the impact of 0-500ng/ml CCN1 on the CB-ECFC proliferation at 24 and 48 hours **(A, B)**. Ki67 expression in CB-ECFC measured by western blot and located by immunofluorescence after stimulated by 0, 100 and 500ng/ml CCN1 **(C–E)**. The migration capacity of CB-ECFCs stimulated by 0-500ng/ml CCN1 using wound healing assay **(F, G)**. The impact of 0-500ng/ml CCN1 on angiogenesis capacity of CB-ECFC at 6 hours **(H)**, characterized by total length **(I)**, branches **(J)** (Green line in ImageJ 6h), numbers of junctions **(K)** (Red ring in ImageJ 6h), meshes **(L)** (Sky blue filled area in ImageJ 6h) and segments **(M)** (Yellow line in ImageJ 6h). Significance for all data was determined by one-way ANOVA analysis tests. Data are presented as mean ± S.D. *p< 0.05, ** p<0.01.

To assess the impact of CCN1 on CB-ECFC proliferation, cells from 5 normal pregnancies were treated with CCN1 (0–500 ng/ml). MTT assays revealed that 100 ng/ml CCN1 significantly decreased proliferation at 48h, and 500 ng/ml CCN1 significantly suppressed proliferation at 24h and 48h (**Figures 3A, B**). These concentrations were selected for further functional analyses. Immunofluorescence and Western blotting showed reduced Ki67 expression (a proliferation marker) in CB-ECFCs treated with 100 and 500 ng/ml CCN1 (**Figures 3C–E**). Wound healing assays showed impaired migration at 500 ng/ml (**Figures 3F, G**). Tube-formation assays further showed that CCN1 reduced vascular network formation, as reflected by decreased total tube length and fewer junctions and branches (**Figures 3H–M**). Together, these results indicate that exogenous CCN1 impairs CB-ECFC proliferation, migration and angiogenic function.

### 3.5 CCN1 accelerates CB-ECFCs senescence through Notch1-mediated oxidative stress and DNA damage

**Figure 4:**
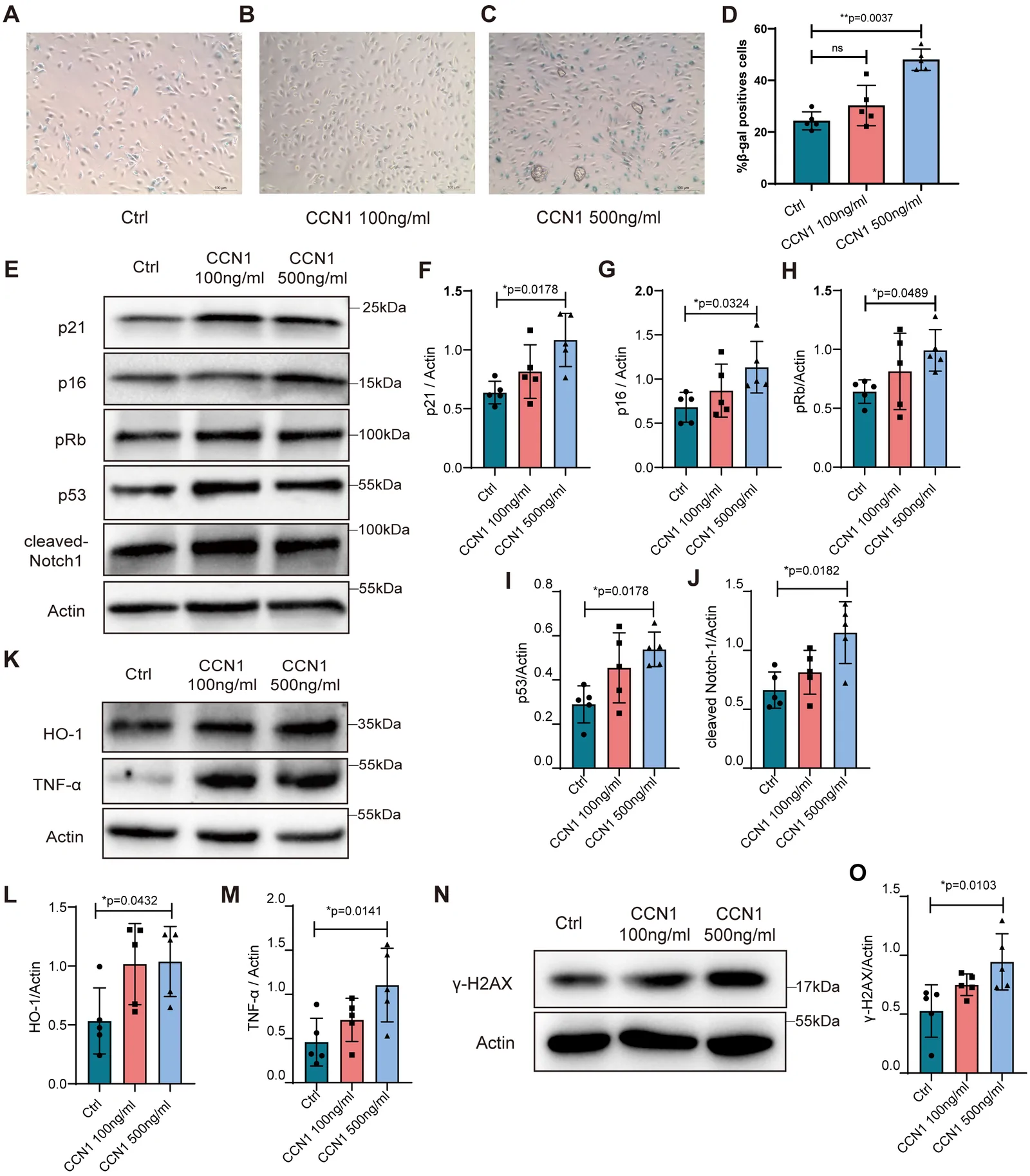
Changes in senescence levels, cleaved-Notch1 expression, oxidative stress and DNA damage of CB-ECFC following treatment with 0–500 ng/ml CCN1. SA-β-Gal stanning for the impact of 0-500ng/ml CCN1 on CB-ECFC **(A–D)**. The expression level of senescence related factors (p16/p21/p53/pRb), cleaved Notch1, oxidative stress related factors (HO-1/TNF-α) and DNA damage related factor (γ-H2AX) of CB-ECFC, after stimulated by CCN1 at 0, 100 and 500ng/ml **(E–M)**. Significance for all data was determined by one-way ANOVA analysis tests. Data are presented as mean ± S.D. *p < 0.05, ** p<0.01.

To explore CB-ECFC dysfunction, cells from healthy pregnancies were treated with increasing concentrations of CCN1 (0, 100, or 500 ng/mL). SA-β-Gal staining demonstrated that 500 ng/mL CCN1 significantly induced senescence (**Figures 4A–D**), consistent with elevated protein levels of the senescence markers p21, p16, pRb, and p53 (**Figures 4E–I**).

Cleaved Notch1 expression was also significantly upregulated at 500 ng/mL CCN1 (p = 0.0182; **Figures 4E, J**). Given the known relationship among Notch1 signaling, oxidative stress, and DNA damage, we assessed the oxidative stress markers HO-1 and TNF-α, as well as the DNA damage marker γ-H2AX. As shown in **Figures 4K–O**, CCN1 treatment increased all three markers. These findings indicate that CCN1-induced CB-ECFC senescence is likely driven by oxidative stress and DNA damage downstream of Notch1 pathway activation.

### 3.5 DAPT attenuates selected CCN1-associated molecular and functional changes

To clarify the role of Notch1 signaling in CCN1-induced CB-ECFC senescence, rescue experiments were performed using DAPT, a γ-secretase inhibitor that suppresses Notch activation. CB-ECFCs were stimulated with 500 ng/mL CCN1, which was selected as the optimal concentration for inducing senescence.

**Figure 5:**
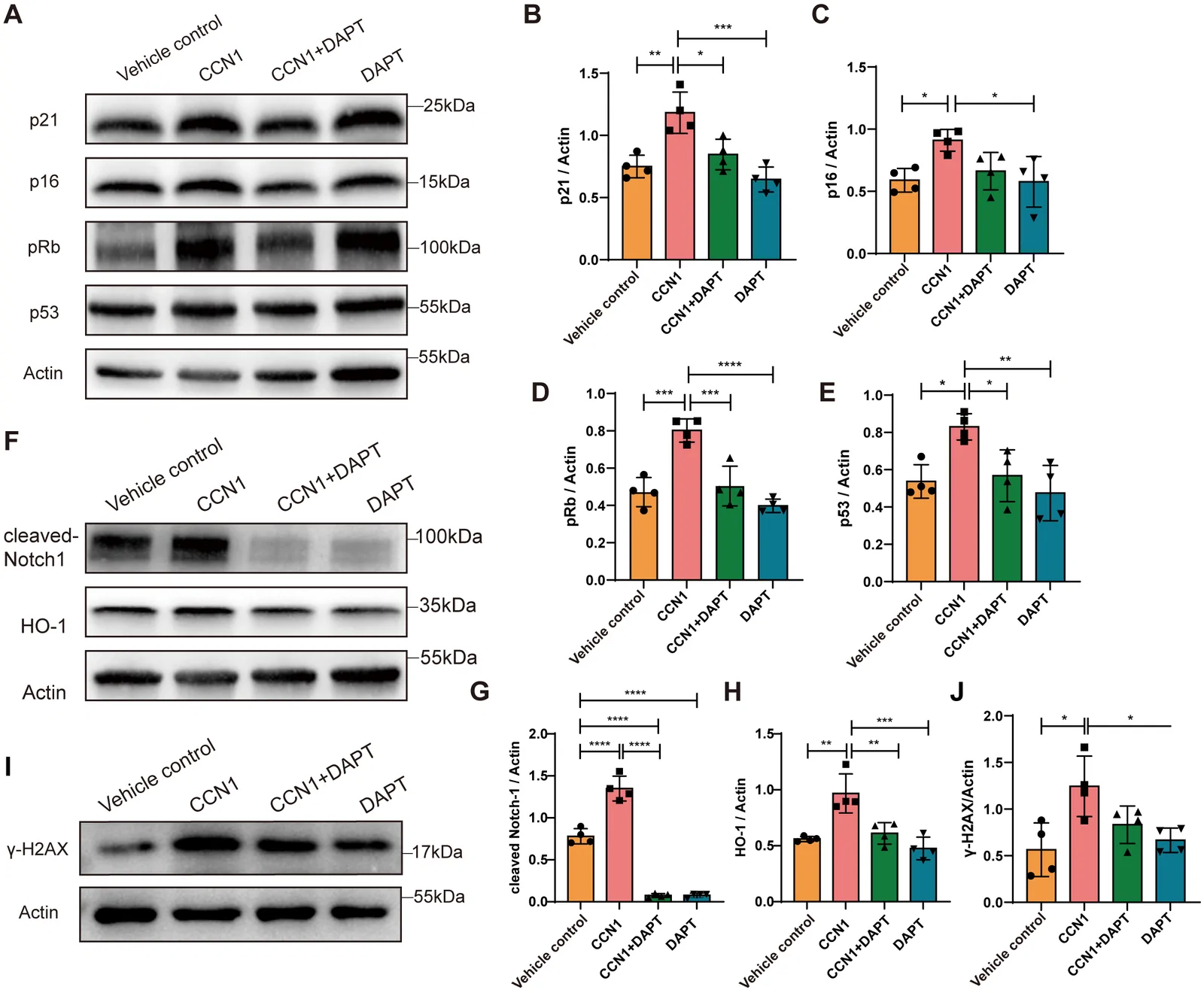
Changes in Notch1, oxidative stress, DNA damage and senescence levels of CB-ECFC following CCN1 treatment (with or without DAPT). CB-ECFCs were divided into four groups: (1) DMSO Vehicle control: Cells were cultured in basic medium with only negligible DMSO to maintain controlled variables; (2) CCN1: Cells were treated with 500ng/ml CCN1 in basic medium; (3) CCN1+DAPT: Cells were co-treated with CCN1 and DAPT in basic medium; (4) DAPT: Cells were treated with DAPT alone in basic medium. The expression levels of p21, p16, pRb, p53, cleaved-Notch1, HO-1 and γ-H2AX were detected by western blot **(A–J)**. Significance for all data was determined by one-way ANOVA analysis tests. Data are presented as mean ± S.D. *p < 0.05, ** p<0.01, ***p<0.001.

SA-β-Gal staining demonstrated that DAPT reduced CCN1-induced senescence in CB-ECFCs (**Supplementary Figure 2**). CCN1 treatment upregulated the expression of senescence-associated markers, including p21, p16, pRb, and p53, whereas these effects were significantly reversed by DAPT treatment (**Figures 5A–E**). Western blotting analysis revealed that cleaved-Notch1 expression was reduced after DAPT treatment (**Figures 5F, G**). Similarly, DAPT reduced the expression of the oxidative stress-related protein HO-1 and the DNA damage marker γ-H2AX compared with CCN1 treatment alone (**Figures 5F, H-J**). These results suggest that CCN1 promotes oxidative stress, DNA damage, and senescence in CB-ECFCs through Notch-associated signaling.

**Figure 6:**
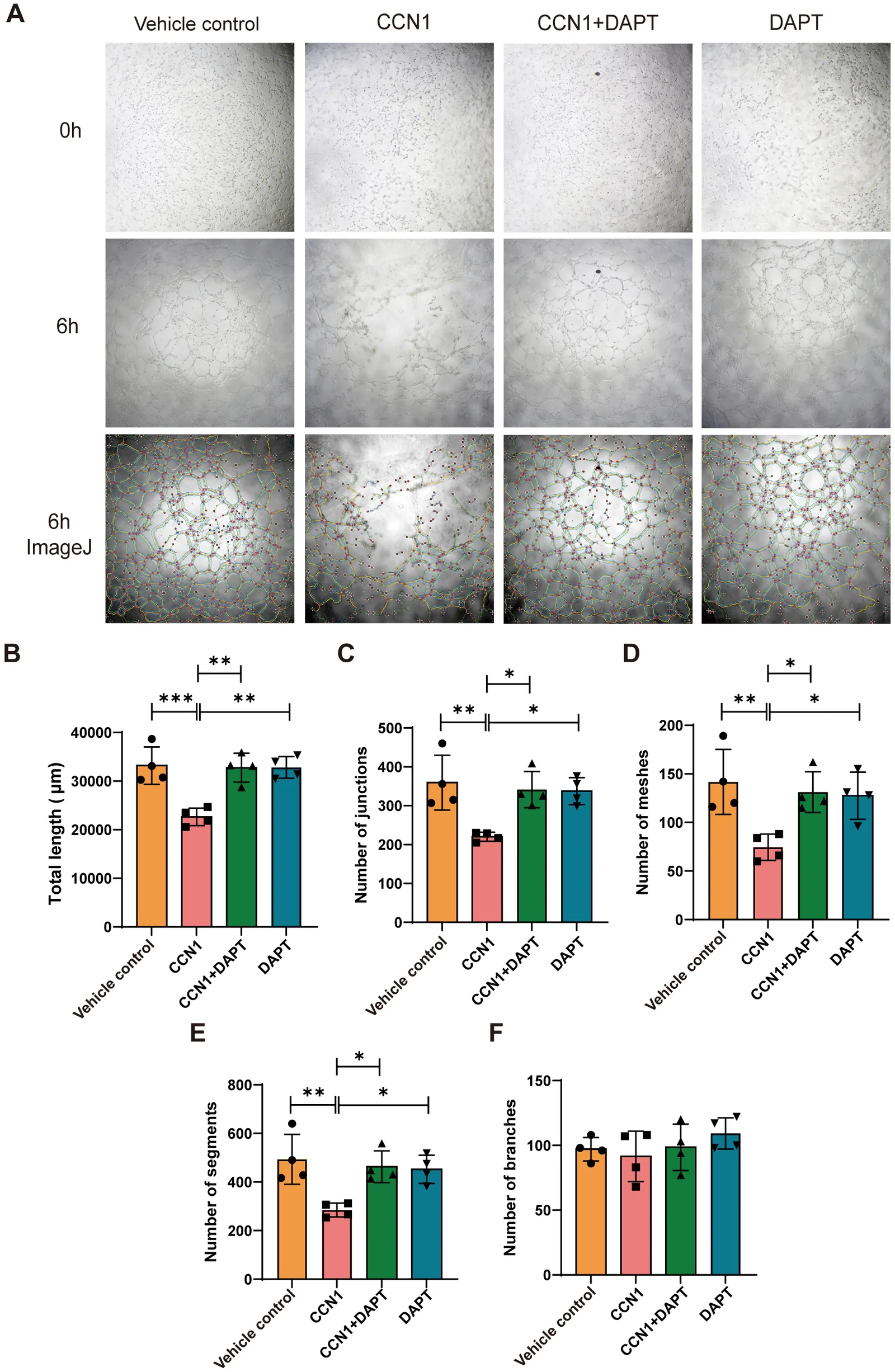
Angiogenesis assay of CB-ECFC following CCN1 treatment (with or without DAPT). CB-ECFCs were divided into four groups: (1) Vehicle control: Cells were cultured in basic medium with only negligible DMSO to maintain controlled variables; (2) CCN1: Cells were treated with 500ng/ml CCN1 in basic medium; (3) CCN1+DAPT: Cells were co-treated with CCN1 and DAPT in basic medium; (4) DAPT: Cells were treated with DAPT alone in basic medium. The angiogenesis capacity of each group at 6 hours **(A)**, characterized by total length **(B)**, numbers of junctions **(C)** (Red ring in ImageJ 6h), meshes **(D)** (Sky blue filled area in ImageJ 6h), segments **(E)** (Yellow line in ImageJ 6h) and branches **(F)** (Green line in ImageJ 6h). Significance for all data was determined by one-way ANOVA analysis tests. Data are presented as mean ± S.D. *p < 0.05, **p<0.01, ***p<0.001.

Functional assays demonstrated that DAPT partially rescued the CCN1-induced reduction in CB-ECFC tube-forming capacity, including total tube length, the number of junctions, meshes, and segments (**Figure 6**). These findings support the mediating role of Notch signaling in CCN1-induced angiogenicdysfunction. However, DAPT did not restore the CCN1-induced impairment of wound closure (**Supplementary Figure 3**). These findings indicate that tube formation and cell migration may respond differently to γ-secretase inhibition, although the underlying mechanism remains unclear.

## 4 Discussion

The identification of circulating EPCs in adult human peripheral blood by Asahara et al. (1997) marked a pivotal advancement in vascular biology and provided a new framework for understanding vascular repair (32). According to Yoder et al. (29), EPCs are also referred to as ECFCs, exhibit a definitive endothelial phenotype, and demonstrate direct vessel integration capabilities that facilitate neoangiogenesis in ischemic models (33). Among ECFC subsets, CB-ECFCs have particularly high proliferative and vasculogenic potential and are therefore relevant to placental vascular development and fetal endothelial repair (7). PE is characterized by maternal vascular dysfunction and impaired placental angiogenesis (34, 35). Previous studies have reported a reduced number and impaired function of ECFCs in PE (36, 37). In agreement with these studies, we also confirmed that CB-ECFC colony numbers were decreased in the PE group. Moreover, PE-derived CB-ECFCs showed increased SA-β-Gal staining, elevated p16, p21, and p53 expression, and increased secretion of the SASP-related proteins MCP-1 and TNF-α (**Figure 1**). These findings suggest that accelerated CB-ECFC senescence may contribute to impaired endothelial repair capacity in PE.

CCN1, a multifunctional matricellular protein of the CCN family, plays pivotal roles in physiological processes including embryonic development, tissue repair, cellular senescence, and angiogenesis (38, 39). Mechanistically, CCN1 induces senescence through interactions with integrin α6β1 and heparan sulfate proteoglycans (40). Its expression is upregulated in the dermis during intrinsic aging and photoaging (41), and exogenous CCN1 can promote fibroblast senescence and SASP activation (42). Additionally, CCN1 triggers G0/G1 cell cycle arrest in trophoblast cells, suppressing proliferation (21). In this study, both membrane-bound and secreted CCN1 levels were increased in CB-ECFCs from PE pregnancies (**Figures 2A–E**).

Immunofluorescence showed that CCN1 was mainly localized at or near the cell membrane (**Figure 2F**), suggesting that CCN1 may have participated in membrane-associated signaling in CB-ECFCs. Functional assays demonstrated that exogenous CCN1 increased senescence markers while reducing cell viability, Ki67 expression, migration, and tube formation (**Figure 3**). These findings indicated that excessive CCN1 may actively promote CB-ECFC dysfunction rather than simply serving as a marker of PE.

Notch is a receptor in a highly conserved signaling pathway that is crucial for development (43). It regulates a variety of biological processes, including cell fate determination, and its mutations are associated with multiple cardiovascular diseases, such as congenital heart disease (44). Upon activation, Notch1 is cleaved to release the Notch1 intracellular domain, which translocates to the nucleus and regulates transcriptional programs involved in cell maintenance, self-renewal, and disease progression (45). Recent work has shown that cerebral ischemia can induce systemic endothelial Notch1 activation and promote endothelial senescence (46). Other studies have reported that extracellular vesicles derived from human adipose tissue and umbilical cord mesenchymal stem cells can alleviate ageing by inhibiting the Notch1 signaling pathway (47). Consistent with previous reports linking CCN1 to Notch signaling, recombinant CCN1 treatment significantly increased cleaved Notch1 levels in CB-ECFCs. DAPT attenuated the associated increases in p16, p21, p53 and pRb, suggesting that γ-secretase-sensitive signaling, potentially involving Notch activation, contributes to the senescence-associated response. However, these pharmacological data do not establish a direct CCN1–Notch1 interaction or a Notch1-specific causal mechanism. Therefore, our findings support a functional association between CCN1 treatment, Notch signaling and CB-ECFC senescence, which requires confirmation through targeted genetic approaches.

Beyond this relationship, our reliance on a pharmacological inhibitor (DAPT) to establish CCN1’s dependence on Notch signaling is consistent with a growing body of evidence describing functional crosstalk between CCN1 and the Notch pathway in several cellular contexts. CCN1 promotes active Notch1 and downstream Sonic hedgehog expression in pancreatic cancer cells through integrin αvβ3-dependent signaling (48). In cholangiocytes, CCN1 engages integrin αvβ5/αvβ3 to activate NF-κB, induce JAG1 and promote NOTCH1 cleavage and NICD1 release; soluble JAG1 rescues the effects of CCN1 depletion, whereas CCN1 cannot bypass JAG1 loss (49). CCN1 also promotes DLL4 expression and RBP-Jκ-dependent Notch transcriptional activity through integrin-mediated signaling in endothelial cells (50). Consistently, studies in intestinal organoids have identified an integrin αvβ3/αvβ5–NF-κB–JAG1–Notch1 axis downstream of CCN1 (51). Collectively, these findings support a context-dependent, predominantly indirect link between CCN1 and Notch signaling. Therefore, our data should be interpreted as demonstrating a functional association rather than direct CCN1–Notch1 binding. Genetic gain- and loss-of-function experiments were technically challenging in primary CB-ECFCs. We therefore used pharmacological inhibition as an alternative approach in the present study. These limitations are important, and future studies using optimized gene-delivery methods, such as nucleofection or adeno-associated viral vectors, are required to establish a causal relationship between CCN1 and Notch1 signaling.

Notch signaling regulates cell migration in a context-dependent manner. Previous studies have shown that DAPT may exert concentration-dependent effects on endothelial migration and sprouting, whereas alterations in Notch1 signaling can influence cell motility by regulating cell adhesion, intercellular junctions, and cytoskeletal organization in different cellular models (52–56). In the present study, DAPT partially restored CCN1-impaired tube formation but did not recover wound closure. These divergent responses suggest that tube formation and migration may involve partly distinct signaling mechanisms. However, because only one DAPT concentration was examined, our findings do not establish a biphasic effect of Notch1 signaling on CB-ECFC migration. Further dose-response experiments and analyses of migration-related downstream targets are required to clarify this issue.

Oxidative stress and DNA damage are well-established contributors to endothelial senescence. HO-1 is an oxidative stress-responsive enzyme involved in the regulation of cellular redox status, while γ-H2AX is widely used as a marker of the cellular response to DNA damage (57–59). CB-ECFCs generally exhibit low senescence under physiological conditions (60), but may be susceptible to stress-induced senescence. In the present study, recombinant CCN1 treatment increased HO-1, TNF-α, and γ-H2AX expression, whereas DAPT attenuated HO-1 and γ-H2AX levels. These molecular changes are compatible with the activation of cellular stress and DNA damage-response pathways during the CCN1-associated senescence phenotype. Our results are in line with previous studies linking oxidative stress-induced cellular senescence, including senescence of endothelial progenitor cells (61, 62). However, HO-1 expression does not provide a direct measurement of intracellular reactive oxygen species, and γ-H2AX alone is insufficient to fully characterize the extent and nature of DNA damage. The precise relationship between Notch1 activation and oxidative stress is likely to be context-dependent, as Notch1 has been reported to exert both pro-oxidative and antioxidant effects in different cellular settings (63, 64). Direct measurement of intracellular ROS, together with assessment of additional DNA damage-response markers such as p-ATM and 53BP1, will be required in future studies to clarify whether oxidative stress and DNA damage mechanistically contribute to the effects of CCN1 in CB-ECFCs.

**Figure 7:**
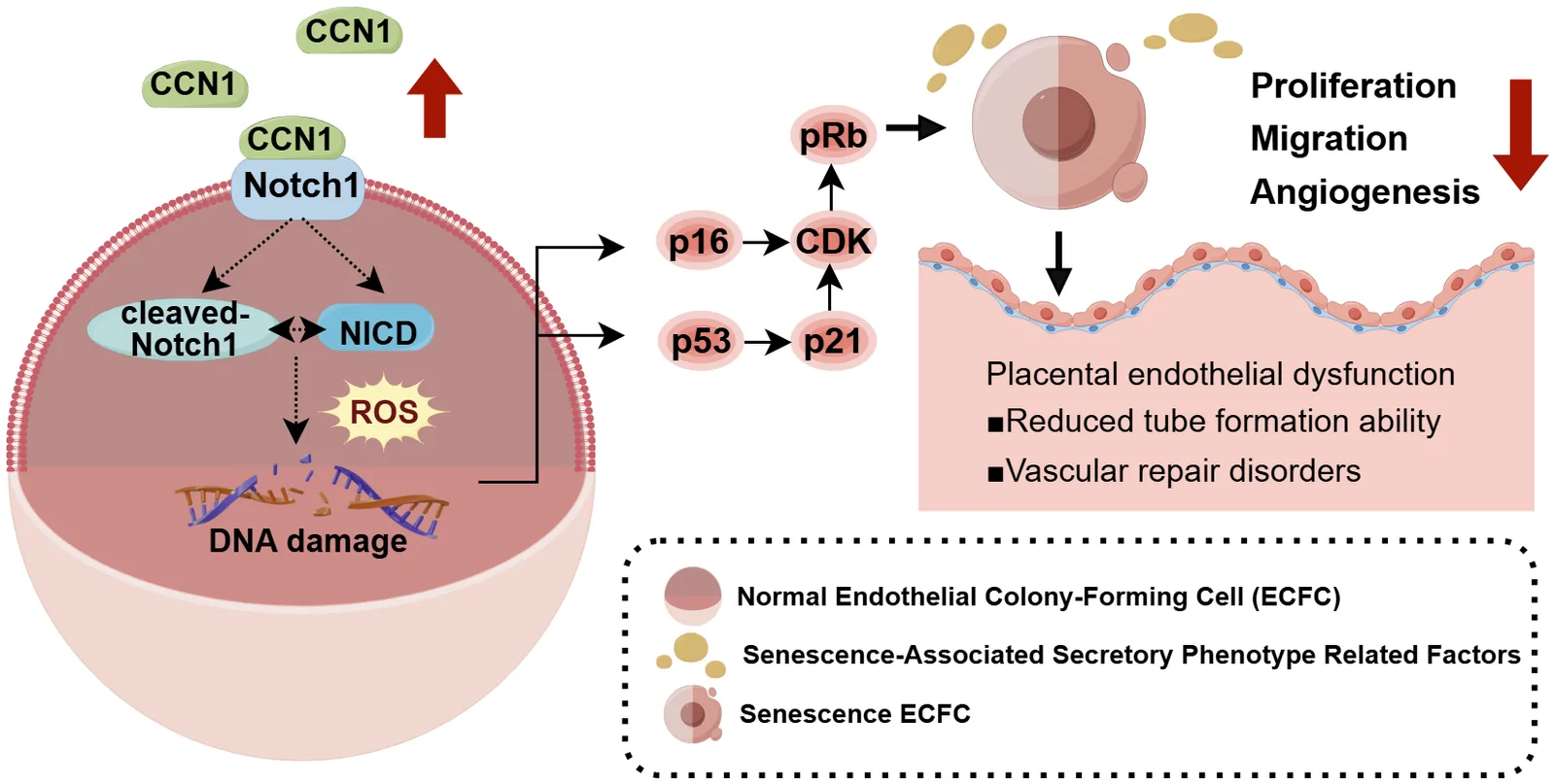
Proposed working model of CCN1-associated signaling in CB-ECFCs. Recombinant CCN1 was associated with increased cleaved Notch1, stress-response and DNA damage-response markers, cellular senescence, and impaired CB-ECFC functions. Dashed arrows indicate proposed relationships that require further genetic, biochemical, and *in vivo* validation.

In summary, our findings identified increased CCN1 expression as a molecular feature of PE-derived cord blood endothelial progenitor cells (CB-ECFCs) and suggest a functional association between CCN1, Notch-related signaling and cellular senescence. *In vitro* treatment with recombinant CCN1 increased cleaved Notch1 and several senescence-, stress- and DNA damage-response markers and was accompanied by impaired CB-ECFC proliferation, migration and tube formation. DAPT partially attenuated these molecular and functional changes, indicating that γ-secretase-sensitive signaling may contribute to the observed response. Collectively, these findings support a working model in which altered CCN1 signaling may be associated with CB-ECFC dysfunction in PE and could potentially compromise endothelial repair and angiogenic capacity(**Figure 7**). However, the present data do not establish a direct CCN1–Notch1 interaction, a Notch1-specific mechanism, or a causal contribution of CB-ECFC dysfunction to placental vascular abnormalities or impaired fetal growth.

This study has several limitations. First, the relatively small sample size, together with the subdivision of control CB-ECFC isolates to different intervention experiments, may have reduced statistical power and limited comparison between experiments. Second, this study was conducted predominantly *in vitro*, and the link between CCN1-associated CB-ECFC senescence and placental vascular remodeling has not yet been confirmed *in vivo*. Third, mechanistic inference was based largely on recombinant CCN1 stimulation and pharmacological γ-secretase inhibition. Genetic gain- and loss-of-function experiments were technically challenging in primary CB-ECFCs: using siRNA to manipulate CCN1 expression in primary CB-ECFC achieved low transfection efficiency (<10%), whereas lentiviral CCN1 overexpression caused substantial loss of viability and spontaneous senescence, precluding reliable functional analysis. Moreover, because DAPT inhibits γ-secretase rather than Notch1 itself; therefore, off-target and cytotoxic effects cannot be ruled out. Finally, HO-1 is an indirect indicator of cellular redox regulation, and intracellular ROS was not directly measured. Although increased γ-H2AX is compatible with activation of a DNA damage response, additional markers such as p-ATM and 53BP1 were not assessed.

In conclusion, CB-ECFCs derived from preeclamptic pregnancies exhibited increased senescence and impaired cellular function, accompanied by increased CCN1 expression. *In vitro* exposure to recombinant CCN1 reproduced several senescence-associated molecular and functional alterations and was accompanied by increased cleaved Notch1 and stress- and DNA damage-response markers. The partial attenuation of these effects by DAPT suggests that γ-secretase-sensitive signaling, potentially involving Notch, may contribute to the response. These findings support a functional association between CCN1, Notch-related signaling and CB-ECFC dysfunction in preeclampsia; however, the underlying causal mechanisms and their relevance to placental endothelial dysfunction require further genetic, biochemical and *in vivo* validation.

## Data Availability

The original contributions presented in the study are included in the article/**Supplementary Material**. Further inquiries can be directed to the corresponding authors.

## References

[1] Hakim J Senterman MK Hakim AM . Preeclampsia is a biomarker for vascular disease in both mother and child: The need for a medical alert system. Int J Pediatr. (2013) 2013:953150. doi: 10.1155/2013/953150 23690796 PMC3652204

[2] Ahmed R Dunford J Mehran R Robson S Kunadian V . Pre-eclampsia and future cardiovascular risk among women: A review. J Am Coll Cardiol. (2014) 63:1815–22. doi: 10.1016/j.jacc.2014.02.529 24613324

[3] de Ganzo Suárez T de Paco Matallana C Plasencia W . Spiral, uterine artery doppler and placental ultrasound in relation to preeclampsia. Best Pract Res Clin Obstet Gynaecol. (2024) 92:102426. doi: 10.1016/j.bpobgyn.2023.102426 38039843

[4] Opichka MA Rappelt MW Gutterman DD Grobe JL McIntosh JJ . Vascular dysfunction in preeclampsia. Cells. (2021) 10. doi: 10.3390/cells10113055 34831277 PMC8616535

[5] Torres-Torres J Espino YSS Martinez-Portilla R Borboa-Olivares H Estrada-Gutierrez G Acevedo-Gallegos S . A narrative review on the pathophysiology of preeclampsia. Int J Mol Sci. (2024) 25. doi: 10.3390/ijms25147569 39062815 PMC11277207

[6] Xu S Ilyas I Little PJ Li H Kamato D Zheng X . Endothelial dysfunction in atherosclerotic cardiovascular diseases and beyond: From mechanism to pharmacotherapies. Pharmacol Rev. (2021) 73:924–67. doi: 10.1124/pharmrev.120.000096 34088867

[7] Melero-Martin JM . Human endothelial colony-forming cells. Cold Spring Harb Perspect Med. (2022) 12. doi: 10.1101/cshperspect.a041154 35379656 PMC9530069

[8] von Versen-Höynck F Brodowski L Dechend R Myerski AC Hubel CA . Vitamin D antagonizes negative effects of preeclampsia on fetal endothelial colony forming cell number and function. PloS One. (2014) 9:e98990. doi: 10.1371/journal.pone.0098990 24892558 PMC4044051

[9] Gao Q Zhu X Chen J Mao C Zhang L Xu Z . Upregulation of P53 promoted G1 arrest and apoptosis in human umbilical cord vein endothelial cells from preeclampsia. J Hypertens. (2016) 34:1380–8. doi: 10.1097/hjh.0000000000000944 27115339 PMC5649442

[10] Beasley KM Lovering AT Gilbert JS . Decreased endothelial progenitor cells in preeclampsia and consequences for developmental programming. Hypertension. (2014) 64:23–5. doi: 10.1161/hypertensionaha.114.03200 24752433 PMC4100588

[11] Monga R Buck S Sharma P Thomas R Chouthai NS . Effect of preeclampsia and intrauterine growth restriction on endothelial progenitor cells in human umbilical cord blood. J Matern Fetal Neonatal Med. (2012) 25:2385–9. doi: 10.3109/14767058.2012.697228 22640270

[12] Gumina DL Su EJ . Endothelial progenitor cells of the human placenta and fetoplacental circulation: A potential link to fetal, neonatal, and long-term health. Front Pediatr. (2017) 5:41. doi: 10.3389/fped.2017.00041 28361046 PMC5350128

[13] van der Feen DE Bossers GPL Hagdorn QAJ Moonen JR Kurakula K Szulcek R . Cellular senescence impairs the reversibility of pulmonary arterial hypertension. Sci Transl Med. (2020) 12. doi: 10.1126/scitranslmed.aaw4974 32727916 PMC7891555

[14] Simoncini S Toupance S Labat C Gautier S Dumoulin C Arnaud L . Functional impairment of endothelial colony forming cells (Ecfc) in patients with severe atherosclerotic cardiovascular disease (Ascvd). Int J Mol Sci. (2022) 23. doi: 10.3390/ijms23168969 36012229 PMC9409296

[15] Toda N . Age-related changes in endothelial function and blood flow regulation. Pharmacol Ther. (2012) 133:159–76. doi: 10.1016/j.pharmthera.2011.10.004 22079549

[16] Go E Tarnawsky SP Shelley WC Banno K Lin Y Gil CH . Mycophenolic acid induces senescence of vascular precursor cells. PloS One. (2018) 13:e0193749. doi: 10.1371/journal.pone.0193749 29538431 PMC5851606

[17] Borghesi A Massa M Campanelli R Bollani L Tzialla C Figar TA . Circulating endothelial progenitor cells in preterm infants with bronchopulmonary dysplasia. Am J Respir Crit Care Med. (2009) 180:540–6. doi: 10.1164/rccm.200812-1949OC 19574444

[18] Kerek R Sawma Awad J Bassam M Hajjar C Ghantous F Rizk K . The multifunctional protein Ccn1/Cyr61: Bridging physiology and disease. Exp Mol Pathol. (2025) 142:104969. doi: 10.1016/j.yexmp.2025.104969 40286773

[19] Qin Z Robichaud P He T Fisher GJ Voorhees JJ Quan T . Oxidant exposure induces cysteine-rich protein 61 (Ccn1) via c-Jun/Ap-1 to reduce collagen expression in human dermal fibroblasts. PloS One. (2014) 9:e115402. doi: 10.1371/journal.pone.0115402 25536346 PMC4275215

[20] Jiang P Ying X Li Z Jiang R Zhou J Zhang M . Hdac5 inactivates Cyr61-regulated Cd31/Mtor axis to prevent the occurrence of preeclampsia. Cell Tissue Res. (2022) 390:281–92. doi: 10.1007/s00441-022-03652-7 35900603

[21] Kipkeew F Kirsch M Klein D Wuelling M Winterhager E Gellhaus A . Ccn1 (Cyr61) and Ccn3 (Nov) signaling drives human trophoblast cells into senescence and stimulates migration properties. Cell Adh Migr. (2016) 10:163–78. doi: 10.1080/19336918.2016.1139265 26744771 PMC4853050

[22] Zhao H Cao N Liu Q Zhang Y Jin R Lai H . Inhibition of the E3 ligase Ubr5 stabilizes Tert and protects vascular organoids from oxidative stress. J Transl Med. (2024) 22:1080. doi: 10.1186/s12967-024-05887-0 39609696 PMC11605888

[23] Yuan R Xu H Wang M Guo L Yao Y Zhang X . Promoting the transition from pyroptosis to apoptosis in endothelial cells: A novel approach to alleviate methylglyoxal-induced vascular damage. J Transl Med. (2025) 23:170. doi: 10.1186/s12967-025-06195-x 39930472 PMC11809013

[24] Levin HI Sullivan-Pyke CS Papaioannou VE Wapner RJ Kitajewski JK Shawber CJ . Dynamic maternal and fetal Notch activity and expression in placentation. Placenta. (2017) 55:5–12. doi: 10.1016/j.placenta.2017.04.014 28623973 PMC5754215

[25] Hasan SS Fischer A . Notch signaling in the vasculature: Angiogenesis and angiocrine functions. Cold Spring Harb Perspect Med. (2023) 13. doi: 10.1101/cshperspect.a041166 35667708 PMC9899647

[26] Liu X Luo Q Zheng Y Liu X Hu Y Liu W . Notch1 impairs endothelial progenitor cell bioactivity in preeclampsia. Reprod Sci. (2017) 24:47–56. doi: 10.1177/1933719116648411 27189202

[27] Meyer N Vu TH Brodowski L Schröder-Heurich B von Kaisenberg C von Versen-Höynck F . Fetal endothelial colony-forming cell impairment after maternal kidney transplantation. Pediatr Res. (2023) 93:810–7. doi: 10.1038/s41390-022-02165-x 35732823 PMC10033415

[28] Grundmann M Haidar M Placzko S Niendorf R Darashchonak N Hubel CA . Vitamin D improves the angiogenic properties of endothelial progenitor cells. Am J Physiol Cell Physiol. (2012) 303:C954–62. doi: 10.1152/ajpcell.00030.2012 22932684 PMC3492823

[29] Ingram DA Mead LE Tanaka H Meade V Fenoglio A Mortell K . Identification of a novel hierarchy of endothelial progenitor cells using human peripheral and umbilical cord blood. Blood. (2004) 104:2752–60. doi: 10.1182/blood-2004-04-1396 15226175

[30] Wood JA Colletti E Mead LE Ingram D Porada CD Zanjani ED . Distinct contribution of human cord blood-derived endothelial colony forming cells to liver and gut in a fetal sheep model. Hepatology. (2012) 56:1086–96. doi: 10.1002/hep.25753 22488442 PMC3396735

[31] Rossi E Poirault-Chassac S Bieche I Chocron R Schnitzler A Lokajczyk A . Human endothelial colony forming cells express intracellular Cd133 that modulates their vasculogenic properties. Stem Cell Rev Rep. (2019) 15:590–600. doi: 10.1007/s12015-019-09881-8 30879244

[32] Asahara T Murohara T Sullivan A Silver M van der Zee R Li T . Isolation of putative progenitor endothelial cells for angiogenesis. Science. (1997) 275:964–7. doi: 10.1126/science.275.5302.964 9020076

[33] Ha G Ferratge S Naserian S Proust R Ponsen A-C Arouche N . Circulating endothelial progenitors in vascular repair. J Cell Immunother. (2018) 4. doi: 10.1016/j.jocit.2018.09.004 38826717

[34] Mannaerts D Faes E Gielis J Van Craenenbroeck E Cos P Spaanderman M . Oxidative stress and endothelial function in normal pregnancy versus pre-eclampsia, a combined longitudinal and case control study. BMC Pregnancy Childbirth. (2018) 18:60. doi: 10.1186/s12884-018-1685-5 29482567 PMC5827979

[35] Chen Y Wan G Li Z Liu X Zhao Y Zou L . Endothelial progenitor cells in pregnancy-related diseases. Clin Sci (Lond). (2023) 137:1699–719. doi: 10.1042/cs20230853 37986615 PMC10665129

[36] Sugawara J Mitsui-Saito M Hayashi C Hoshiai T Senoo M Chisaka H . Decrease and senescence of endothelial progenitor cells in patients with preeclampsia. J Clin Endocrinol Metab. (2005) 90:5329–32. doi: 10.1210/jc.2005-0532 15956081

[37] Hwang HS Maeng YS Park YW Koos BJ Kwon YG Kim YH . Increased senescence and reduced functional ability of fetal endothelial progenitor cells in pregnancies complicated by preeclampsia without intrauterine growth restriction. Am J Obstet Gynecol. (2008) 199:259.e1–7. doi: 10.1016/j.ajog.2008.06.060 18771975

[38] Kim KH Won JH Cheng N Lau LF . The matricellular protein Ccn1 in tissue injury repair. J Cell Commun Signal. (2018) 12:273–9. doi: 10.1007/s12079-018-0450-x 29357009 PMC5842204

[39] Zhu Y Almuntashiri S Han Y Wang X Somanath PR Zhang D . The roles of Ccn1/Cyr61 in pulmonary diseases. Int J Mol Sci. (2020) 21. doi: 10.3390/ijms21217810 33105556 PMC7659478

[40] Lau LF . Ccn1/Cyr61: The very model of a modern matricellular protein. Cell Mol Life Sci. (2011) 68:3149–63. doi: 10.1007/s00018-011-0778-3 21805345 PMC3651699

[41] Yokota M Kamiya Y Suzuki T Ishikawa S Takeda A Kondo S . Trehangelins ameliorate inflammation-induced skin senescence by suppressing the epidermal Yap-Ccn1 axis. Sci Rep. (2022) 12:952. doi: 10.1038/s41598-022-04924-6 35046484 PMC8770704

[42] Feng T Meng J Kou S Jiang Z Huang X Lu Z . Ccn1-induced cellular senescence promotes heart regeneration. Circulation. (2019) 139:2495–8. doi: 10.1161/circulationaha.119.039530 31107624

[43] Bray SJ . Notch signalling: A simple pathway becomes complex. Nat Rev Mol Cell Biol. (2006) 7:678–89. doi: 10.1038/nrm2009 16921404

[44] Meester JAN Verstraeten A Alaerts M Schepers D Van Laer L Loeys BL . Overlapping but distinct roles for Notch receptors in human cardiovascular disease. Clin Genet. (2019) 95:85–94. doi: 10.1111/cge.13382 29767458

[45] Rice MA Hsu EC Aslan M Ghoochani A Su A Stoyanova T . Loss of Notch1 activity inhibits prostate cancer growth and metastasis and sensitizes prostate cancer cells to antiandrogen therapies. Mol Cancer Ther. (2019) 18:1230–42. doi: 10.1158/1535-7163.Mct-18-0804 31028097 PMC7280023

[46] Liu M Wang D Qi C Zou M Song J Li L . Brain ischemia causes systemic Notch1 activity in endothelial cells to drive atherosclerosis. Immunity. (2024) 57:2157–2172.e7. doi: 10.1016/j.immuni.2024.07.002 39079536

[47] Zhang H Xiao X Wang L Shi X Fu N Wang S . Human adipose and umbilical cord mesenchymal stem cell-derived extracellular vesicles mitigate photoaging via Timp1/Notch1. Signal Transduct Target Ther. (2024) 9:294. doi: 10.1038/s41392-024-01993-z 39472581 PMC11522688

[48] Haque I De A Majumder M Mehta S McGregor D Banerjee SK . The matricellular protein Ccn1/Cyr61 is a critical regulator of Sonic Hedgehog in pancreatic carcinogenesis. J Biol Chem. (2012) 287:38569–79. doi: 10.1074/jbc.M112.389064 23027863 PMC3493902

[49] Kim KH Chen CC Alpini G Lau LF . Ccn1 induces hepatic ductular reaction through integrin Avβ_5_-mediated activation of Nf-Kb. J Clin Invest. (2015) 125:1886–900. doi: 10.1172/jci79327 25822023 PMC4463205

[50] Chintala H Krupska I Yan L Lau L Grant M Chaqour B . The matricellular protein Ccn1 controls retinal angiogenesis by targeting Vegf, Src homology 2 domain phosphatase-1 and Notch signaling. Development. (2015) 142:2364–74. doi: 10.1242/dev.121913 26002917 PMC4510592

[51] Won JH Choi JS Jun JI . Ccn1 interacts with integrins to regulate intestinal stem cell proliferation and differentiation. Nat Commun. (2022) 13:3117. doi: 10.1038/s41467-022-30851-1 35660741 PMC9166801

[52] Shi Q Xue C Zeng Y Yuan X Chu Q Jiang S . Notch signaling pathway in cancer: From mechanistic insights to targeted therapies. Signal Transduct Target Ther. (2024) 9:128. doi: 10.1038/s41392-024-01828-x 38797752 PMC11128457

[53] Liu T Li W Zhang J Zhang Y . Mir-222-3p inhibits trophoblast cell migration and alleviates preeclampsia in rats through inhibiting Hdac6 and Notch1 signaling. Reprod Sci. (2022) 29:1486–97. doi: 10.1007/s43032-021-00793-y 34796469

[54] Zhang Y Qin X Guo R Sun X Zhao Z Guo H . Notch-1 regulates collective breast cancer cell migration by controlling intercellular junction and cytoskeletal organization. Cell Prolif. (2025) 58:e13754. doi: 10.1111/cpr.13754 39343985 PMC11839191

[55] Cao L Arany PR Wang YS Mooney DJ . Promoting angiogenesis via manipulation of Vegf responsiveness with Notch signaling. Biomaterials. (2009) 30:4085–93. doi: 10.1016/j.biomaterials.2009.04.051 19481797 PMC2730921

[56] Bolós V Mira E Martínez-Poveda B Luxán G Cañamero M Martínez AC . Notch activation stimulates migration of breast cancer cells and promotes tumor growth. Breast Cancer Res. (2013) 15:R54. doi: 10.1186/bcr3447 23826634 PMC3978930

[57] Johmura Y Yamanaka T Omori S Wang TW Sugiura Y Matsumoto M . Senolysis by glutaminolysis inhibition ameliorates various age-associated disorders. Science. (2021) 371:265–70. doi: 10.1126/science.abb5916 33446552

[58] Biswas C Shah N Muthu M La P Fernando AP Sengupta S . Nuclear heme oxygenase-1 (Ho-1) modulates subcellular distribution and activation of Nrf2, impacting metabolic and anti-oxidant defenses. J Biol Chem. (2014) 289:26882–94. doi: 10.1074/jbc.M114.567685 25107906 PMC4175329

[59] Li C Wu J Dong Q Ma J Gao H Liu G . The crosstalk between oxidative stress and DNA damage induces neural stem cell senescence by Ho-1/Parp1 non-canonical pathway. Free Radic Biol Med. (2024) 223:443–57. doi: 10.1016/j.freeradbiomed.2024.07.020 39047850

[60] Zhang Y Herbert BS Rajashekhar G Ingram DA Yoder MC Clauss M . Premature senescence of highly proliferative endothelial progenitor cells is induced by tumor necrosis factor-alpha via the P38 mitogen-activated protein kinase pathway. FASEB J. (2009) 23:1358–65. doi: 10.1096/fj.08-110296 19124561 PMC2669419

[61] von Zglinicki T . Oxidative stress and cell senescence as drivers of ageing: Chicken and egg. Ageing Res Rev. (2024) 102:102558. doi: 10.1016/j.arr.2024.102558 39454760

[62] Reskiawan AKR Alwjwaj M Ahmad Othman O Rakkar K Sprigg N Bath PM . Inhibition of oxidative stress delays senescence and augments functional capacity of endothelial progenitor cells. Brain Res. (2022) 1787:147925. doi: 10.1016/j.brainres.2022.147925 35469846

[63] Kang JW He JP Liu YN Zhang Y Song SS Xu QX . Aberrant activated Notch1 promotes prostate enlargement driven by androgen signaling via disrupting mitochondrial function in mouse. Cell Mol Life Sci. (2024) 81:155. doi: 10.1007/s00018-024-05143-0 38538986 PMC10973062

[64] Li Y Guo A Liu J Tang L Su L Liu Z . Myeloid-specific knockout of Notch-1 inhibits Myd88- and Trif-mediated Tlr signaling pathways by regulating oxidative stress-Shp2 axis, thus restraining aneurysm progression. Aging (Albany NY). (2024) 16:1182–91. doi: 10.18632/aging.205392 38284891 PMC10866402

