# Supplementary material for: CCN1-induced senescence and dysfunction of umbilical cord blood endothelial colony-forming cells in the pathogenesis of preeclampsia
Source: Front Endocrinol (Lausanne). 2026 Aug 28;17:1930702. doi: 10.3389/fendo.2026.1930702 (PMC13561788; doi:10.3389/fendo.2026.1930702)
Supplement: Supplementary file 1 [file SupplementaryFile1.docx]

Supplementary Material

# 1 Supplementary Tables

| **Table S1. List of primers for qPCR in this study** | | | |
| --- | --- | --- | --- |
| **Gene** | **NCBI accession number** | **Primer Sequence (5’ → 3’)** | **Product length** |
| p16 | [XM_011517676](https://www.ncbi.nlm.nih.gov/entrez/viewer.fcgi?db=nucleotide&id=1034663488) | for: CATGGAGCCTTCGGCTGAC  rev: GGCCTCCGACCGTAACTATT | 120 bp |
| p53 | [NM_001276761.3](https://www.ncbi.nlm.nih.gov/entrez/viewer.fcgi?db=nucleotide&id=1889531459) | for:CCTCAGCATCTTATCCGAGTGG  rev:TGGATGGTGGTACAGTCAGAGC | 128bp |
| p21 | [NM_001291549.2](https://www.ncbi.nlm.nih.gov/entrez/viewer.fcgi?db=nucleotide&id=1677537872) | for:GCGATGGACTTCGACTTTG  rev:CAGGTCCACATGGTCTTCCT | 198 bp |
| CCN1 | [NM_001554.5](https://www.ncbi.nlm.nih.gov/entrez/viewer.fcgi?db=nucleotide&id=1519312167) | for: GCAGCGTTTCCCTTCTACAG  rev: CACCCTTGGCAGTTTCGAAA | 223bp |
| Actin | [NM_001101.5](https://www.ncbi.nlm.nih.gov/entrez/viewer.fcgi?db=nucleotide&id=1519311456) | for:CACCATTGGCAATGAGCGGTTC  rev: AGGTCTTTGCGGATGTCCACGT | 135 bp |

| **Table S2. List of primary, secondary antibodies and dilution factors for western blotting in this study** | | | | | |
| --- | --- | --- | --- | --- | --- |
| **Primary antibody** | **Host** | **Primary antibody** | | **Second antibody** | |
|  |  | **company** | **concentration** | **Company** | **concentration** |
| MCP-1 | rabbit | Cell Signaling(#23236) | 1:1000 | Abcam(ab6721)  goat anti-rabbit | 1:5000 |
| TNF-α | rabbit | HUABIO(HA722022) | 1:1000 | Abcam(ab6721)  goat anti-rabbit | 1:5000 |
| CCN1 | rabbit | Cell Signaling(#14479) | 1:1000 | Abcam(ab6721)  goat anti-rabbit | 1:5000 |
| Ki67 | rabbit | Sigma(SAB5700770) | 1:1000 | Abcam(ab6721)  goat anti-rabbit | 1:5000 |
| p21 | rabbit | Cell Signaling (#2947S) | 1:750 | Abcam(ab6721)  goat anti-rabbit | 1:5000 |
| p16 | rabbit | Abcam (ab108349) | 1:250 | Abcam(ab6721)  goat anti-rabbit | 1:2500 |
| p53 | rabbit | Cell Signaling (#9284S) | 1:1000 | Abcam(ab6721)  goat anti-rabbit | 1:5000 |
| pRb | rabbit | Cell Signaling (#9307S) | 1:1000 | Abcam(ab6721)  goat anti-rabbit | 1:5000 |
| cleaved Notch-1 | rabbit | Cell Signaling (#4147T) | 1:250 | Abcam(ab6721)  goat anti-rabbit | 1:3500 |
| HO-1 | rabbit | Sigma(SAB5700731) | 1:1000 | Abcam(ab6721)  goat anti-rabbit | 1:5000 |
| γ-H2AX | mouse | Abcam(ab81299) | 1:1000 | Abcam(ab205719)  Goat anti-mouse | 1:5000 |
| β-Actin | mouse monoclonal | Sigma(A3854) (HRP-conjugated) | 1:25,000 | / | / |

| Table S3. List of primary, secondary antibodies for immunofluorescence in this study | | |
| --- | --- | --- |
| **Primary antibody** | **Host (clone)** | **Company** |
| CD31 | mouse monoclonal | Abcam(ab9498) |
| CCN1 | rabbit monoclonal | Cell Signaling(#14479) |
| Ki67 | rabbit polyclonal | Sigma(SAB5700770) |
| **Secondary antibody** | **Host (clone)** | **Company** |
| Alexa Fluor 488 | donkey anti rabbit IgG | Life Technologies (R37118) |
| Cy3 | goat anti mouse | Life Technologies (A10521) |

# 2 Supplementary Figures

## 2.1 Supplementary Figure 1

**Supplementary Figure 1.** Characterization of CB-ECFCs. (A-C) Cells were analyzed for expression of CD31 (A), CD45 (B) and CD133 (C); (D) Immunofluorescence stanning for the location and expression of CD31 in CB-ECFCs.

## 2.2 Supplementary Figure 2

**Supplementary Figure 2.** Changes in senescence levels of CB-ECFCs following CCN1 treatment (with or without DAPT). CB-ECFCs were assigned to four experimental groups: (1) Vehicle control: cells cultured in basal medium containing a low concentration of DMSO as vehicle control; (2) CCN1: cells treated with 500 ng/mL CCN1 in basal medium; (3) CCN1 + DAPT: cells co-incubated with CCN1 and DAPT in basal medium; (4) DAPT: cells treated with DAPT alone in basal medium. (A,B) SA-β-Gal staining and quantitative analysis for the four groups, cells positive for SA-β-Gal were stained blue, representing senescent cells. Significance for all data was determined by one-way ANOVA analysis tests. Data are presented as mean±S.D. ***p<0.001, ****p<0.0001.

CCN1+DAPT

Vehicle control

CCN1

DAPT

**AA**

**BA**

## 2.3 Supplementary Figure 3

0h

**H**

8h

**H**

DAPT

CCN1+DAPT

CCN1

**AA**

**BA**

Vehicle control

**Supplementary Figure 3.** Migration ability of CB-ECFCs upon CCN1 stimulation with or without DAPT co-treatment. CB-ECFCs were assigned to four experimental groups: (1) Vehicle control: cells cultured in basal medium containing a low concentration of DMSO as vehicle control; (2) CCN1: cells treated with 500 ng/mL CCN1 in basal medium; (3) CCN1 + DAPT: cells co-incubated with CCN1 and DAPT in basal medium; (4) DAPT: cells treated with DAPT alone in basal medium. (A, B) Wound healing migration assay of CB-ECFCs from the four groups. (A) Representative wound closure images; (B) Quantitative statistical analysis of wound healing rates. Statistical significance was assessed via one-way ANOVA. All data are expressed as mean ± S.D. *p < 0.05, **p < 0.01, ***p < 0.001.
